# Role of heparanase in ARDS through autophagy and exosome pathway (review)

**DOI:** 10.3389/fphar.2023.1200782

**Published:** 2023-06-08

**Authors:** Fei Feng, Lin-Jun Wang, Jian-Chun Li, Ting-Ting Chen, Liping Liu

**Affiliations:** ^1^ The First Clinical Medical School of Lanzhou University, Lanzhou, China; ^2^ Departments of Emergency Critical Care Medicine, The First Hospital of Lanzhou University, Lanzhou, Gansu, China

**Keywords:** heparinase, ARDS, exosome, inflammation, coagulation, fibrosis, autophagy

## Abstract

Acute respiratory distress syndrome (ARDS) is the most common respiratory disease in ICU. Although there are many treatment and support methods, the mortality rate is still high. The main pathological feature of ARDS is the damage of pulmonary microvascular endothelium and alveolar epithelium caused by inflammatory reaction, which may lead to coagulation system disorder and pulmonary fibrosis. Heparanase (HPA) plays an significant role in inflammation, coagulation, fibrosis. It is reported that HPA degrades a large amount of HS in ARDS, leading to the damage of endothelial glycocalyx and inflammatory factors are released in large quantities. HPA can aggrandize the release of exosomes through syndecan-syntenin-Alix pathway, leading to a series of pathological reactions; at the same time, HPA can cause abnormal expression of autophagy. Therefore, we speculate that HPA promotes the occurrence and development of ARDS through exosomes and autophagy, which leads to a large amount of release of inflammatory factors, coagulation disorder and pulmonary fibrosis. This article mainly describes the mechanism of HPA on ARDS.

## 1 Introduction

Acute respiratory distress syndrome (ARDS) is an acute respiratory failure characterized by pulmonary edema, progressive dyspnea and refractory hypoxemia caused by increased alveolar capillary permeability (Thompson et al., 2017). The prevalence of ARDS in intensive care units (ICU) in 50 countries is 10.4% (Murray et al., 2019). Although ARDS has been widely studied, its mortality rate is still as high as 34.9%–46% (Zhang et al., 2017). In recent years, many COVID-19 cases have developed into ARDS (Batah and Fabro, 2021).

There are a lot of inducing factors for the occurrence and development of ARDS, including direct lung injury (bacterial and viral pneumonia, inhalation of gastric contents and pulmonary contusion) and indirect extrapulmonary injury (sepsis, severe trauma, blood transfusion, pancreatitis, drug reaction etc.). Once these inducing factors occur, the pathophysiology of ARDS will be manifested in the complex interaction between the inflammatory, coagulation, immune system and the alveolar capillary barrier, which will eventually lead to fiber proliferation (Hughes and Beasley, 2017). It includes the following three aspects: (1) Acute inflammatory response (Simeonovic et al., 2013): increased permeability of alveolar capillaries, injury of alveolar epithelial cells, decreased secretion of pulmonary surfactant, impaired clearance of alveolar fluid, increased expression of adhesion molecules, extravasation of white blood cells and their released products, immune system disorder, and finally tissue destruction; (2) Coagulation disorder (Freeman and Parish, 1998): intravascular coagulation, fibrin deposition and hyperfibrinolysis; (3) Fibrosis: regeneration of alveolar endothelial cells and epithelial type I/II cells, fiber proliferation (Fernandez-Francos et al., 2021). So far, despite the progress in understanding the biology, physiology and pathology of ARDS, there is no effective drug for the treatment of ARDS, and its treatment scheme is mainly limited to supportive therapy (Neff et al., 2003). Therefore, there is a need for pioneering methods to achieve effective treatment of ARDS.

Heparanase (HPA) is the only functional endoglycosidase capable of cleaving heparan sulfate (HS) chains (Vlodavsky et al., 1999). HPA specifically induces the degradation of HS in glycocalyx, thereby aggravating the injury of pulmonary endothelial barrier (Chappell et al., 2008) and the increase of alveolar permeability^12^, leading to the formation of ARDS (Coulson-Thomas et al., 2015; Chen et al., 2017), affecting the recovery of lung injury (LaRiviere et al., 2020). HPA also has non enzymatic functions (Secchi et al., 2015), such as regulating gene expression, promoting cell adhesion (Nadir, 2014; Sanderson et al., 2017). HPA increased the release of exosomes and caused abnormal expression of autophagy. It has been reported that there is a relationship between the development of ARDS and exosomes, autophagy (Yamada, 2021). Therefore, we searched “ARDS, heparanase, inflammation, immune, coagulation, fibrosis, exosome, autophagy” in electronic databases such as “PubMed” and “Web of Science,” to explore the mechanism of HPA participating in the occurrence and development of ARDS.

## 2 Heparanase

HPA is an endonuclease-β-D-glucuronidase, the only endoglycosidase to degrade basement membrane extracellular matrix (ECM) and heparan sulfate glycoprotein (Vlodavsky et al., 2007). The gene expression of HPA can be regulated by early growth response genes in tumor cells (de Mestre et al., 2005), inflammatory cytokines and fatty acids in endothelial cells (Chen et al., 2004). The human HPA gene is located on chromosome 4q22 and has two splice variants (Dong et al., 2000). Its cDNA (complementary deoxyribonucleic acid) contains an open reading frame of 1629 base pairs. Cleavage of the N-terminal signal peptide generates a 65kda inactive HPA precursor (Vreys and David, 2007).

According to the effect of HPA on heparin and HS, the enzymes are divided into three categories: HPA I (heparin lyase I), which mainly acts on heparin; HPA II (heparin lyase II), acting on heparin and HS; HPA III (heparin lyase III) mainly acts on HS (Yamada and Sugahara, 1998; Hu et al., 2015). HPA I is known to destroy specific binding points between glucosamine residues (called s-domain), where sulfate groups bind to uronic acid (Bame, 2001; Gingis-Velitski et al., 2004; Reiland et al., 2004). HPA III can cleave bioactive HS fragments by degrading HS (Kato et al., 1998).

The physiological expression of HPA is limited to a few cell and tissue types, such as platelets, immune cells and placenta (Vlodavsky et al., 1992; Goshen et al., 1996; Hulett et al., 1999; Gutter-Kapon et al., 2016; Putz et al., 2017). In adults, HPA may play a role in wound repair, tissue regeneration and immune monitoring. HPA plays a role in cell adhesion, migration and survival (Goldshmidt et al., 2003). HPA can affect the migration of inflammatory cells, inflammatory cell specific cytokine interferon-γ (IFN-γ) and tumor necrosis factor (TNF) can stimulate endothelial cells to produce HPA and enhance the activity of HPA (Bartlett et al., 1995; Edovitsky et al., 2006; Ilan et al., 2006), destroy the cell barrier by degrading HS (Simeonovic et al., 2013), causes a series of inflammatory reactions. Therefore, HPA can cause inflammatory reaction under pathological conditions, leading to a series of reactions in the body.

HPA has both enzymatic and non enzymatic activities. HPA selectively cleaves HS polymers to produce fragments of variable size, usually 10–20 sugar units, indicating that only a limited proportion of glucuronic acid bonds in the HS chain are vulnerable to the enzyme (Goldshmidt et al., 2003). HPA plays a role by releasing growth factors from ECM (Ilan et al., 2006), participates in the degradation and remodeling of extracellular matrix, promotes cell invasion related to inflammation metastasis (Myler and West, 2002). The cleavage of HS seems to be the key for leukocytes to pass through the basement membrane (Finkel, 1999a; Bame, 2001). Its activity is related to inflammatory acidosis (McKenzie et al., 2003). Its enhanced activity can also regulate macrophage activation (cytokine expression induced by c-fos) (Parish, 2006), promotes the recruitment of immune cells to the injury site (remodeling through extracellular matrix) (Shteingauz and Vlodavsky, 2015) and enhances the neuroinflammatory effect of autophagy (Dai et al., 2014). Non enzymatic HPA can induce endothelial cell invasion and migration through PI3K/Akt pathway (Gingis-Velitski et al., 2004; Yuan et al., 2012). Non enzymatic HPA also enhance T cell adhesion, mediated by integrin β (Sotnikov et al., 2004). After the non enzymatic active HPA is activated through PKA (protein kinase A) and PKC (protein kinase C) signaling pathways, the lysosome secretes the active form of HPA (Shafat et al., 2006).

## 3 Heparanase promotes ARDS through inflammation and immune disorders

The pathogenesis of ARDS is complex, and the disorder of inflammatory regulation plays an important role (Matthay et al., 2019). In the early stage of ARDS, microvascular endothelial cells are first affected and are less resistant to injury than epithelial cells (Wiener-Kronish et al., 1991; Matthay et al., 2019). Extensive damage of endothelial cell (EC) barrier and inflammatory cascade lead to increased permeability, which is the core pathogenesis of ARDS (Wiener-Kronish et al., 1991; Han and Mallampalli, 2015). Macrophages in the lung secrete a large number of proinflammatory factors, leading to the recruitment of macrophages and activation of effector T cells. These processes further exacerbate the inflammatory response and tissue damage patients with ARDS (Aggarwal et al., 2014; Long et al., 2019; Park et al., 2019), an inflammatory response called “cytokine storm” may lead to multiple organ failure and increase patient mortality (Chappell et al., 2008). With the secretion of inflammatory factors, cytokines increase abnormally in other tissues and organs, interfere with the immune system, cause excessive immunity, and lead to diffuse lung cell injury, pulmonary fibrosis and multiple organ injury (Zhang et al., 2021a). The innate immune response is initially triggered by pulmonary epithelial cells, alveolar macrophages and neutrophils (Rokni et al., 2020). In mucosal immune response, interleukin-22 (IL-22) upregulates mucin, fibrinogen and anti-apoptotic protein; therefore, IL-22 may contribute to the formation of life-threatening edema, and the lungs may be rich in mucin and fibrin, leading to the progress of ARDS (Wu and Yang, 2020). When the immune system is reduced by lymphocytes, it may be one of the mechanisms leading to the development of ARDS (Yazdanpanah et al., 2020). The substances in ARDS may have a binding and signal transduction mode similar to that of lung tissue cells, and may be responsible for the recruitment and activation of corresponding immune cells and acute lung injury (Lee, 2017). Therefore, the occurrence and development of ARDS are related to the aggravation of inflammation and immune disorders.

HPA can affect the migration of inflammatory cells and destroy the cell barrier by degrading HS (Simeonovic et al., 2013). Inflammatory cell specific cytokine IFN-γ and TNF can stimulate endothelial cells to produce HPA and enhance the activity of HPA (Bartlett et al., 1995; Edovitsky et al., 2006; Ilan et al., 2006). Therefore, HPA derived from endothelial cells can also promote the migration of lymphocytes and granulocytes, macrophages and dendritic cells through bone marrow (BM) and ECM under endothelium (Parish et al., 2013). HPA produced by leukocytes can be induced by various cell activation stimuli (de Mestre et al., 2003; Chen et al., 2004; Putz et al., 2017), promoting leukocyte migration (Poon et al., 2014; Digre et al., 2017), cell rolling and adhesion (Lever et al., 2014; Changyaleket et al., 2017), proinflammatory factors are upregulated (Goodall et al., 2014) and activation of innate immune cells (Gutter-Kapon et al., 2016). In colitis, epithelial derived HPA regulates the inflammatory phenotype of macrophages, prevents inflammation from fading, and converts macrophage responses to chronic inflammatory patterns (Lerner et al., 2011). HPA also enhanced macrophage activation *in vitro* through lipopolysaccharide (LPS) and increased TNF-α, IL-6 and IL-12. Activated macrophages in turn can induce epithelial HPA expression and promote self-sustaining inflammatory circuits through increased secretion of cathepsin-l (Lerner et al., 2011). HPA is involved in the recruitment of pulmonary inflammatory factors in allergic asthma models (Morris et al., 2015). HPA causes the degradation of endothelial glycocalyx in sepsis associated lung injury, which aggregates neutrophils and inflammatory factors (Schmidt et al., 2012), causing a series of inflammatory reactions. Inhibition of HPA activity can prevent endotoxemia related loss of pulmonary endothelial glycocalyx, thus alleviating sepsis induced inflammatory lung injury in mice (Schmidt et al., 2012). In sepsis related intestinal injury, HPA inhibitors prevent the destruction of glycocalyx of intestinal mucosa, inhibit neutrophil infiltration, protect mucosal integrity, and inhibit inflammatory response by inhibiting HPA activity (Chen et al., 2017). Heparin and heparin derived compounds can compete with HS chain to bind HPA and inhibit the activity of HPA (Goldshmidt et al., 2004; Waterman et al., 2007), so they are potent HPA inhibitors (Bar-Ner et al., 1987). It plays an anti-inflammatory role in the treatment of asthma, patients undergoing extracorporeal circulation and cataract surgery (Mousavi et al., 2015).

Therefore, HPA maybe promote the activity of inflammatory cells and immune disorders, increases the aggregation of neutrophils, degrades HS in the glycocalyx of lung endothelium, and causes the permeability of alveolar vascular wall to increase, thus leading to ARDS. But the specific mechanism is still unclear, further discussion is needed.

## 4 Heparanase promotes ARDS through coagulation

Thrombosis and coagulation disorder maybe the main factors leading to ARDS (Helms et al., 2020). However, the underlying mechanism remains unclear. Tissue factor (TF) can be produced by endothelial cells, smooth muscle cells, neutrophils and monocytes to respond to various stimuli *in vivo* and *in vitro* (DelGiudice and White, 2009; Kambas et al., 2012), which is a key link in the process of coagulation *in vivo* (Rapaport and Rao, 1995; Mackman et al., 2007). Platelets are the key to thrombosis. During blood transfusion, activated platelets can induce acute lung injury (Caudrillier et al., 2012). In recent corona virus disease 2019 (COVID-19) pathology, activated platelets from patients showed the production of TF bearing nets, inducing thrombotic activity of human aortic endothelial cells (HAECs) (Skendros et al., 2020). Experiments have proved that (Zhang et al., 2021b) in the plasma of patients with ARDS, the TF expression of neutrophils is significantly increased and reticular cells are exposed. Thrombin is reported to be necessary for protease activated receptor-1 (PAR-1) to activate platelets (Petzold et al., 2020). Thrombin activated platelets can increase the formation of TF network and subsequent immune thrombosis in patients with ARDS (Zhang et al., 2021b). With the support of immune cells, platelets and coagulation related molecules, immune thrombosis is considered to be a key event in the pathophysiology of ARDS. When sepsis mediated ARDS occurs, neutrophils are activated and then form TF rich neutrophil extracellular traps in pulmonary vessels (the first step), resulting in thrombin production; Platelets are activated by thrombin and then interact with neutrophils to form TF networks (step 2). All these factors lead to a vicious circle leading to a large number of thrombosis (Zhang et al., 2021b). Therefore, the development of ARDS may be closely related to coagulation.

Under normal conditions, HPA activity is limited to placental and skin tissues, as well as blood cells, such as neutrophils, monocytes, mast cells, T lymphocytes and platelets. Among them, platelets have the highest HPA activity and are used as a source of activated HPA (Freeman and Parish, 1998; Freeman et al., 1999). There is evidence that HPA may also affect the coagulation system (Matzner et al., 1985; El-Assal et al., 2001; Koliopanos et al., 2001). HPA may be a co-factor of TF and directly participate in the activation of coagulation factors (Nadir et al., 2010). Increased HPA regulates the expression of coagulation factor TF13 and interacts with TFPI (tissue factor pathway inhibitor) on the cell membrane surface of endothelial cells and tumor cells, resulting in the dissociation of TFPI, thereby increasing the coagulation activity on the cell surface (El-Assal et al., 2001). In addition, HPA can directly enhance TF activity, increase the production of factor Xa, and then activate the coagulation system (Rohloff et al., 2002). Over expression of HPA in human leukemia, glioma and breast cancer cells leads to a significant increase in TF level. HPA may promote angiogenesis by inducing the expression of angiogenesis promoting factor (VEGF) and reducing the expression of angiogenesis inhibitory (thrombospondin) mediators by TF (Zhang et al., 1994; Abe et al., 1999). Discovery that natural anticoagulant heparin can inhibit the spread of cancer in animals (Finkel, 1999b). It can prevent platelets from coagulating around cancer cells, and even if the anticoagulant activity of heparin is exhausted, it can still inhibit tumor metastasis (Eccles, 1999), so heparin inhibits HPA activity by competing with HS to bind heparin or HS binding domain (HBD) (Levy-Adam et al., 2005).

In conclusion, the occurrence of ARDS is related to the disorder of the coagulation system, and HPA leads to the disorder of the coagulation system. Therefore, HPA may cause the occurrence and development of ARDS through the disorder of the coagulation system. However, there is no specific report that HPA can cause ARDS through coagulation disorder, so further study is needed.

## 5 Heparanase promotes ARDS through fibrosis

Tissue fibrosis is an unregulated wound healing response characterized by gradual accumulation and reduced remodeling of ECM (Rockey et al., 2015a). In organs such as heart, lung, kidney or liver, the accumulation of fibrous tissue can gradually change its normal structure and function, and may cause destructive results (Rockey et al., 2015b; Jun and Lau, 2018). The main pathogenesis of pulmonary fibrosis is that the injury of alveolar epithelial cells activates lung fibroblasts and promotes their transformation into matrix producing myofibroblasts. Replacing normal lung parenchyma with fibrotic tissue will lead to irreversible reduction of oxygen diffusion capacity (Sakai and Tager, 2013). The acute exudative inflammation stage of ARDS is followed by the proliferation stage characterized by the proliferation of alveolar epithelial cells (Matthay et al., 2019), some ARDS survivors will further develop fibroblast proliferation responses, including fibroblast aggregation, deposition of collagen and other pulmonary ECM components (Burnham et al., 2014). In the ARDS animal model, type II alveolar epithelial intracellular stress is regulated by inducing abnormal mucin expression (Hancock et al., 2018), increased endoplasmic reticulum (ER) stress (Lawson et al., 2011), induce local tissue hypoxia (Xi et al., 2017; Burman et al., 2018), impairing the normal repair process and aggravating the fibrotic response (Michalski et al., 2022). It has recently been reported that high levels of heat shock protein 90 (HSP90) play an important role in the development of pulmonary fibrosis (Sontake et al., 2017). HSP90 is a highly expressed molecular chaperone protein that is important for the physiological function of human cells (Wu et al., 2018). HSP90 is critical in the treatment of lung injury (Sibinska et al., 2017; Marinova et al., 2020). HSP90 has a proinflammatory role in the development and progression of ARDS, and inhibition of HSP90 can maintain pulmonary endothelial integrity (Barabutis and Siejka, 2020) and anti-inflammatory effects (Barabutis et al., 2018). HSP90 inhibitors can promote protein ubiquitination and protease degradation, and significantly improve pulmonary fibrosis (Marinova et al., 2020; Colunga Biancatelli et al., 2021). Therefore, pulmonary fibrosis is the final development result of ARDS, but its specific mechanism has not been clarified, so finding the specific mechanism of pulmonary fibrosis is an important target to improve its prognosis.

The involvement of HPA in fibrosis depends on the fact that HPA promotes the release and diffusion of various HS linked molecules, rather than the catalytic activity responsible for cutting HS side chains (Masola et al., 2020). HPA is a key regulator of fibroblast growth factor-basic 2 (FGF-2) and transfroming growth factor-β (TGF-β) activities, and is a major pro-fibrotic factor and inducer of kidney (Masola et al., 2012; Masola et al., 2014). The increase of HPA activity at the renal tubular level can regulate the epithelial to mesenchymal transition (EMT) of proximal renal tubular cells, thus forming a profibrotic environment (Secchi et al., 2017), lack of HPA can prevent the overexpression of TGF- β (Masola et al., 2014), HPA deficient mice did not show TGF-α increased without fibrosis (Gil et al., 2012), HPA regulates TGF-β by releasing syndecan-1, and the upregulation of TGF-β is associated with intestinal fibrosis *in vivo* (Davids et al., 2010). In non cancerous tissues, HPA expression was negatively correlated with fibrosis stage (Ikeguchi et al., 2003). HPA levels were elevated in the fibrotic livers of thioacetamide treated rats (Goldshmidt et al., 2004; Ohayon et al., 2008). HPA activity in the plasma of patients with mild and severe liver fibrosis increased, while HPA activity in the plasma of patients with cirrhosis decreased to the basal level (Secchi et al., 2017). High mobiliby group box 1 (HMGB1) can activate fibroblasts to myofibroblasts and activate NF through its receptor RAGE-κB and increased HPA expression. Upregulated HPA releases TGF stored in ECM by decomposing HS-β, thus promoting the progress of pulmonary fibrosis (He et al., 2016). At the same time, a related protein also plays a related role in pulmonary fibrosis in ARDS. Matrix metalloproteinases (MMPs) are zinc-dependent endopeptidases whose main role is to degradw collagen and ECM components and can also act on cell surface proteins (Zinter et al., 2019). Elevated plasma MMP-3 levels in sepsis patients are associated with endothelial damage and impaired oxygenation in ARDS (Zinter et al., 2019). MMPs not only play an important role in physiological tissue remodeling and wound repair (Page-McCaw et al., 2007; Hadler-Olsen et al., 2011), but also are associated with pathological processes such as rheumatoid arthritis, cancer, liver, kidney, heart and lung fibrosis (Zuo et al., 2002; Green et al., 2003; Heymans et al., 2005; Uchinami et al., 2006; Rosas et al., 2008). MMP-3, MMP-7 and MMP-9 can all promote the formation of pulmonary fibrosis (Craig et al., 2015). At present, the relationship between HPA and MMP is still unclear, and whether they interact with each other in the occurrence and development of ARDS needs further exploration.

In conclusion, ARDS eventually develops into pulmonary fibrosis, and HPA leads to pulmonary fibrosis. Therefore, HPA may lead to the development of ARDS through pulmonary fibrosis. However, there is no specific report on HPA causing ARDS through fibrosis, so further research is needed.

## 6 Heparanase may affect ARDS through exosomes and autophagy

ARDS is a destructive clinical syndrome characterized by non cardiogenic pulmonary edema, respiratory distress and hypoxemia (Murray et al., 2019). Autophagy is a multi-step dynamic process in cells: damaged or dysfunctional proteins and organelles are separated by double membrane vesicles and fused with lysosomes to form autolysosomes for degradation and recycling (Klionsky et al., 2008; Barth et al., 2010). Under physiological conditions, autophagy is expressed at a low level, which is essential to maintain the stability of the intracellular environment (Sanderson et al., 2017). However, autophagic disorders were observed in different lung diseases, including lung injury (Chang et al., 2015; Chu et al., 2018) and pulmonary fibrosis (Meng et al., 2019). Light chain 3 (LC3) is a major regulator of autophagy formation (Sou et al., 2006). In lung diseases, the penetration of *acinetobacter* baumannii activates autophagy, which is caused by the Beclin1 dependent AMPK-ERK-mTOR pathway (Liao et al., 2019). MSCs may alleviate LPS induced ALI by downregulating miR-142a-5p to enable pulmonary epithelial cells (PECs) to undergo beclin mediated autophagy (Zhou and You, 2016). The most important cell types involved in the pathogenesis of ARDS are macrophages, epithelial cells and neutrophils (Saidi et al., 2018). During the occurrence of ARDS, the relationship between these cell types is mutual influence and interaction, and one cell type “indicates” that another cell type changes its phenotype, while the other type refutes in turn. In the microenvironment (alveolar lavage fluid BALF), the information transmitted between these cells is assembled into some small goods, such as exosomes, which are released from one cell into BALF and then obtained by another cell to decode signals (Lee, 2016) (Soni et al., 2016). Exosomes are nano double lipid membranes secreted by cells in the secretory body, with a diameter of 30–100nm, which are generated in large cells with multiple vesicles (Jun and Lau, 2018). Bidirectional communication occurs in microenvironment through exosomes and microbubbles (MVs) (Stahl and Raposo, 2019). Exosomes carry nucleic acids, proteins and lipids between different cells in the tumor microenvironment, which affects many ways in biology. Bone marrow mesenchymal stem cells (MSCs) are increasingly used to treat ARDS and sepsis because of their immunomodulatory and regenerative properties (Walter et al., 2014). MSC can also inhibit proinflammatory cytokines secretion, thereby potentially relieving the subsequent cytokine storm (Ye et al., 2020). In fact, preliminary preclinical and clinical results show that bone marrow mesenchymal stem cells can alleviate lung dysfunction in animal lung injury models (Curley et al., 2012), ARDS and patients with neocoronal pneumonia (Zheng et al., 2014; Xiao et al., 2020). Bone marrow derived exosomes are a new, multi-target next-generation biological agent, which may be the key to downregulate cytokine storm and reverse host antiviral defense inhibition characterized by neocoronal pneumonia (Hessvik and Llorente, 2018). The exosomes contain a whole set of chemokines, growth factors, mRNA and microRNAs with anti-inflammatory, regenerative and immune regulatory functions. They are paracrine and endocrine mediators, which endow BMSCs with healing characteristics (De Jong et al., 2014; Yu et al., 2014; Alipoor et al., 2016; Hessvik and Llorente, 2018). The exosomes secreted by bone marrow mesenchymal stem cells (BMSCs) are a new therapy for ARDS, but the mechanism between them and HPA is still unclear.

HPA exists in autophagy and promotes autophagy (Shteingauz et al., 2015). The level of LC3II was found to be decreased in cells and tissues obtained from HPA knockout mice, while the level of LC3-II was found to be increased in transgenic mice overexpressing HPA (Shteingauz et al., 2015). The mechanism of HPA induced autophagy is not completely clear, but it may involve mTOR1 (Dunlop and Tee, 2014). Overexpression of HPA is associated with decreased mTOR1 activity (Shteingauz et al., 2015). Electron microscope analysis of cells overexpressing HPA showed that not only more autophagic vacuoles were found, but also a large number of vesicles were released on the cell surface, which may be exosomes (Thompson et al., 2013; Roucourt et al., 2015). HPA localizes to the surface of exosomes secreted by various cell type (Sanderson et al., 2017; Nawaz et al., 2018). HPA affects the composition of protein and mRNA in exosomes, participates in mediating the secretion and function of exosomes, and enhances the secretion of exosomes (Thompson et al., 2013). HPA stimulates endosomal budding of syntenin and syndecan, and Alix is required to achieve these effects. Therefore, HPA is an activator of the syndecan-syntenin-Alix pathway of exosome biogenesis (Roucourt et al., 2015). In myeloma cells, HPA stimulates the accumulation of syndecan-1 and specific cargoes such as hepatocyte growth factor and VEGF in exosomes (Thompson et al., 2013). Recently, HPA has also been involved in the autophagy process. Exosomes and autophagy are connected through the endolysosomal pathway, and there is a strong interaction between them (Xu et al., 2018). HPA exists in autophagy and promotes autophagy, making HPA overexpressing cells more resistant to stress and chemotherapy. The mechanism of increased autophagy is not fully understood, but may involve decreased mTOR1 activity (Shteingauz et al., 2015; Sanderson et al., 2017).

In conclusion, HPA can increase the release of exosomes and promote autophagy. However, the specific mechanism of ARDS is still unclear. Therefore, exploring the specific mechanism between HPA and exosomes, autophagy and ARDS can become an important way to treat ARDS.

## 7 Summary

**FIGURE 1:**
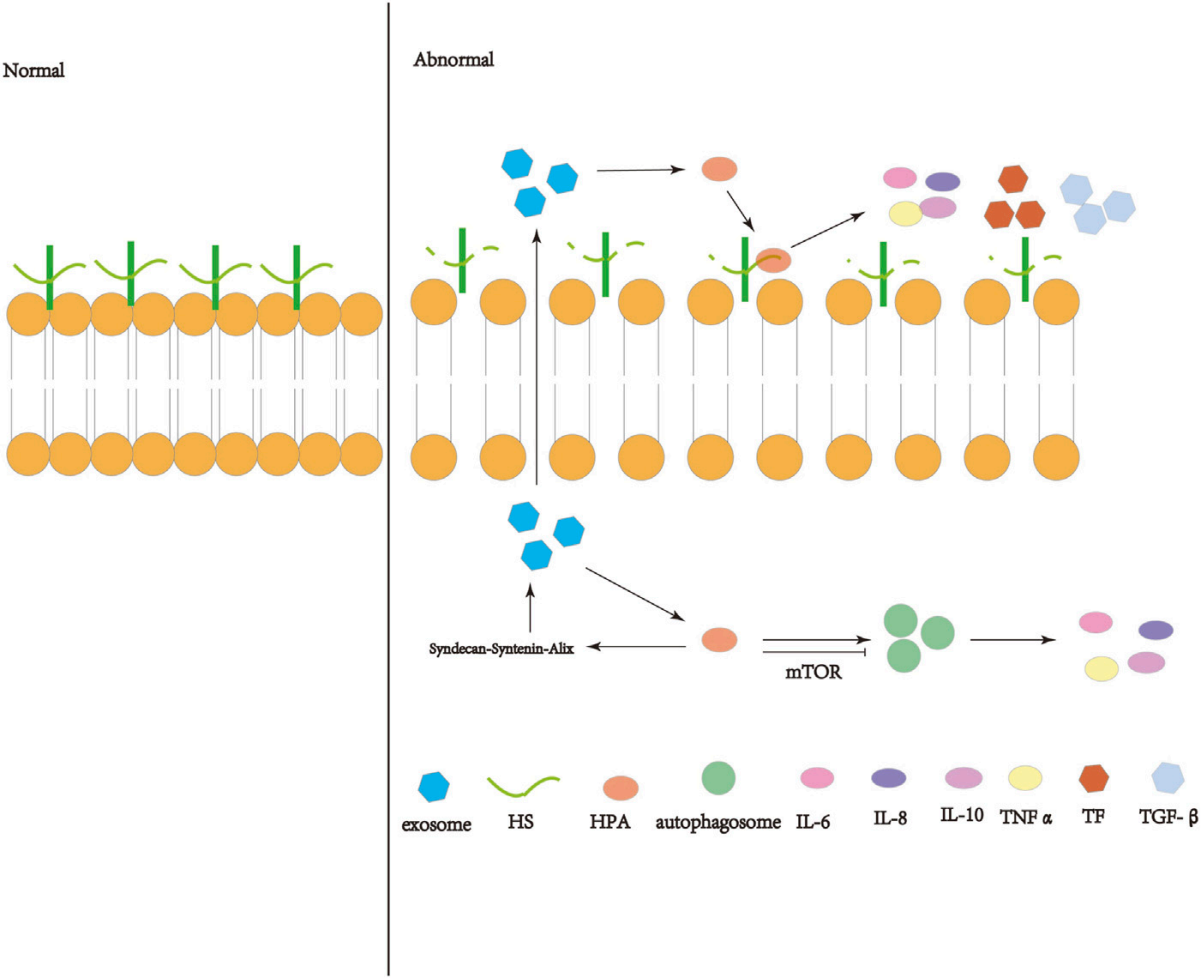
Schematic diagram of the operation and action of heparanase (HPA). HPA can interact with HSPG on the cell membrane, shear the heparin sulfate (HS) side chain, destroy the extracellular matrix (ECM) and basement membrane (BM), and release inflammatory immune factors, coagulation factors, etc. Active HPA can enhance autophagy through the mTOR pathway and increase exosome formation through the syndecan-syntenin-Alix pathway, so that exosomes are released into the extracellular space and act on the extracellular HPA.

With the increase of research on HPA, the influence of HPA in disease is increasing. This review summarizes that HPA can aggravate inflammation, immune system disorder, coagulation dysfunction and tissue fibrosis, which may play a significant role in the occurrence and development of ARDS through inflammation, immune disorder, coagulation disorder and fibrosis. However, the specific mechanism needs to be further explored. At the same time, we speculate that HPA may affect the occurrence and development of ARDS through some signaling pathway of exosomes or autophagy, which needs further study. Therefore, HPA may affects the occurrence and development of ARDS, which may become a new idea to reduce the mortality of ARDS. (Figure 1).
